# Strength Training Characteristics of Different Loads Based on Acceleration Sensor and Finite Element Simulation

**DOI:** 10.3390/s21020647

**Published:** 2021-01-19

**Authors:** Bo Pang, Zhongqiu Ji, Zihua Zhang, Yunchuan Sun, Chunmin Ma, Zirong He, Xin Hu, Guiping Jiang

**Affiliations:** 1 School of Physical Education and Sports, Beijing Normal University, Beijing 100875, China; 201831070010@mail.bnu.edu.cn (B.P.); tggzzh@163.com (Z.Z.); 201922070009@mail.bnu.edu.cn (Z.H.); 201831070016@mail.bnu.edu.cn (X.H.); 01064@bnu.edu.cn (G.J.); 2 Business School, Beijing Normal University, Beijing 100875, China; yunch@bnu.edu.cn; 3 School of Mechanical Engineering and Applied Electronic Technology, Beijing University of Technology, Beijing 100124, China; machunmin@bjut.edu.cn

**Keywords:** sensors, resistance training, deep squat, bench press, hard pull

## Abstract

Deep squat, bench press and hard pull are important ways for people to improve their strength. The use of sensors to measure force is rare. Measuring strength with sensors is extremely valuable for people to master the intensity of exercise to scientifically effective exercise. To this end, in this paper, we used a real-time wireless motion capture and mechanical evaluation system of the wearable sensor to measure the dynamic characteristics of 30 young men performing deep squat, bench press and hard pull maneuvers. The data of tibia were simulated with AnyBody 5.2 and ANSYS 19.2 to verify the authenticity. The result demonstrated that the appropriate force of the deep squat elbow joint, the hip joint and the knee joint is 40% 1RM, the appropriate force of the bench press is 40% 1RM and the appropriate force of the hard pull is 80% 1RM. The external force is the main factor of bone change. The mechanical characteristics of knee joint can be simulated after the Finite Element Analysis and the simulation of AnyBody model are verified.

## 1. Introduction

At present, wearable devices have been widely used in mass fitness and competitive sports [1], and the devices can monitor the training load, competition quality and injury recovery of athletes. Additionally, these devices can scientifically and effectively detect the kinematical indexes of the exercisers, can correct the wrong actions in real-time, and can standardize the movement technology. Wearable equipment is a new product of modern science and technology, with the devices possessing good quality, low weight, a strong ability to function for long time periods without electricity and a reduction in the verification of performed actions. Moreover, the application of wearable devices with fine-tune sensors in strength training can allow for more suitable exercise load plans for athletes [2]. Additionally, the evaluation of the teaching effects on students through the use of wearable devices has a certain guiding significance for allowing for timely feedback [3]. In regard to strength training, scientific research in the aspect of biomechanics has been more developed in foreign countries [4], and further in-depth research has also been conducted in China. Squat, bench press and hard pull have become a significant focus of biomechanical research [5]. The purpose of this study was to test the strength factors of squat, bench press and hard pull, in order to provide a scientific basis and theoretical reference for strength training.

## 2. Materials and Methods

### 2.1. Participants

Thirty-eight male college students were recruited as the subjects. The morphological data of the subjects were collected in various dimensions. Furthermore, the mean age of the subjects was 21.52 ± 0.95-years-old, the mean height was 1.75 ± 0.05 m, the mean BMI (Body Mass Index) was 21.73 ± 1.80, the mean waist value was 78.35 ± 5.40 cm, and the waist-to-hip ratio was 0.79 ± 0.04. The test was completed at the same morning for all subjects to avoid fatigue in the afternoon. The warming up activity consisted of jogging and stretching the muscles of the upper and lower limbs for a low-intensity exercise, which ensured that the muscles were in a state of natural relaxation during the test. The test process was completed by the same tester, 30 subjects completed the test.

### 2.2. Experimental Process

#### 2.2.1. Data Acquisition

**Figure 1:**
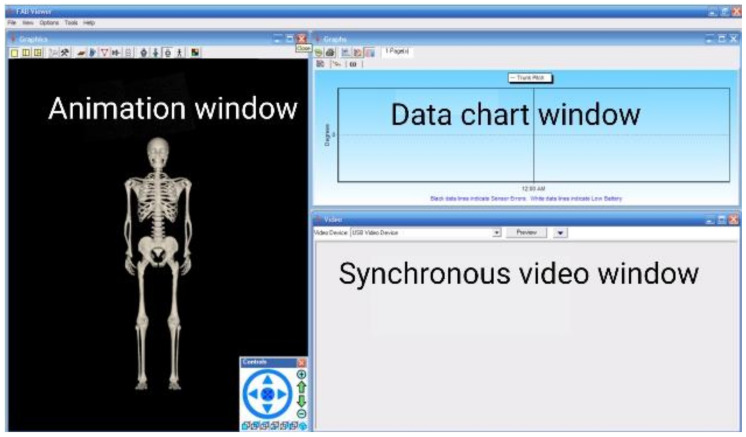
Software analysis interface.

The FAB (Functional Assessment of Biomechanics) real-time wireless motion capture and mechanical evaluation system is produced in Canada. It is a motion evaluation system based on wireless inertial sensing technology. It can also be separated from the limitations of cameras and can be detached from a fixed site, possesses a wireless transmission range of up to 20 m with self-contained storage equipment. Furthermore, data can be stored in a memory card, with no loss of data, and a complete real-time acquisition and display of the captured person’s action measurement data through the use of wireless transmission to computer analysis software, the values of the different load intensity test data’s come from the sensor output (Figure 1). Finally, the synchronous analysis and comparison of all of the real-time data can be conducted, sensing output is impedance, after which the data can be exported into Microsoft Excel and into the motion capture general file format BVH (Biovision Hierarchy motion file).

**Figure 2:**
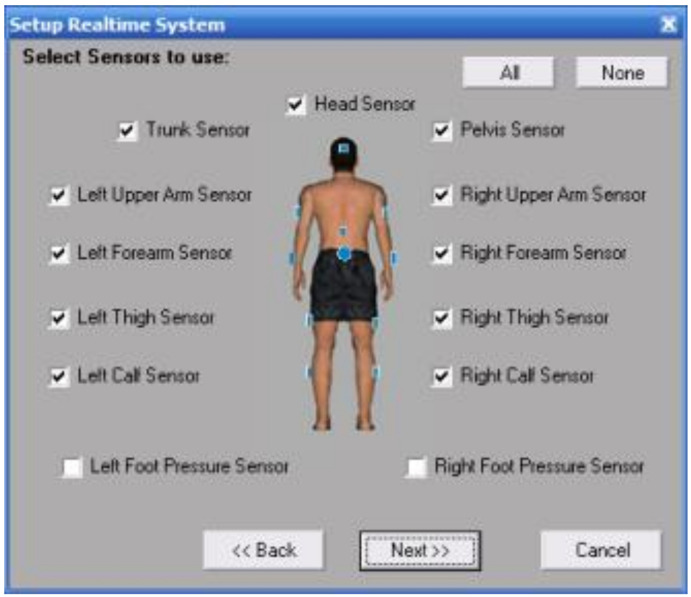
The working interface of the sensor.

The sampling rate of the gyroscope was adjusted to 100 Hz, and the acceleration was adjusted to 100 Hz. Furthermore, the magnetometer was adjusted to 25 Hz, and the angular resolution was set to 0.05 degrees in the FAB wireless real-time mode. The USB port was connected to the computer, the data were collected and transmitted to a small receiver at a sampling frequency of 100 Hz to capture the angles, angular velocities, angular accelerations, forces, torques and power data of the subjects. The video signal was synchronously collected. Additionally, the video duration was no more than 10 min, and the wireless transmission between the signal and the computer was realized (Figure 2). The system was able to play back the captured data and to evaluate the effects of exercise.

The FAB system was composed of an 11 lead standard configuration of small and light sensors. The size of the sensor was 67 × 41 × 25 mm, and the weight was 60 g. The sensors were fixed to each part of the subject with elastic bandages, and after which the parameters of the subject in the neutral position were calibrated.

**Figure 3:**
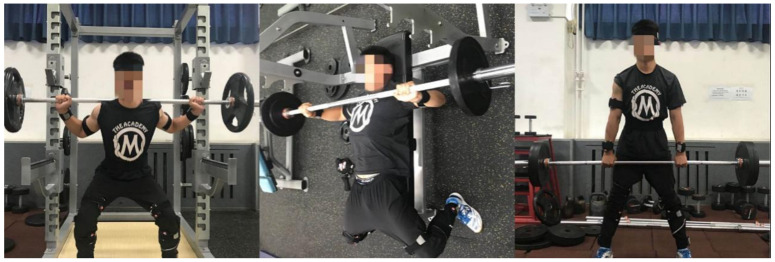
Subject information collection chart.

One repetition maximum testing (1RM) is most often determined via direct assessment of the maximal weight which an individual can lift for one repetition [6]. 1RM refers to the resistance of a person to repeat an action with the correct action. For example, the heaviest bench press of an athlete can lift 100 kg, and can only be lifted once, and its 1RM is 100 kg. In the strength test, 30%–80% was the appropriate load. Each subject filled in the weight of their specific 1RM when they completed the informed consent form. 1RM (maximum load) ≈ω/[1.0278 − (0.0278 * r)] [7], with ω being the total weight of the load, r being the repeated weight under the load. According to Hughes et al. [8], 40%, 60% and 80% 1RM are actually feasible intensities in strength training, 40%, 60% and 80% of the predetermined 1RM load weights were selected for the experiment. After 20 s of rest, moderate force was applied to the individual’s maximum perception of 60% load weight. After 20 s of rest, the individual’s maximum perception of 80% load weight was applied with severe force. The subjects completed the test according to the sequence and content (Figure 3).

#### 2.2.2. AnyBody Simulation Operation

The software for data processing is AnyBody 5.2, which is used to process the muscle strength after FAB action capture. The software has been verified for many times, with strong reliability and high accuracy. Static algorithm of inverse dynamics for AnyBody Modeling System [9,10]:

(1) $$\begin{array}{l} \underset{f}{\mathrm{Minimize}\;}G(f^{\left(M\right)})\\ \mathrm{subject}\;\mathrm{to}\;\\ \mathrm{Cf}=r\\ f_{i}^{\left(M\right)}\geq 0,i\in \left\{1,....n^{\left(M\right)}\right\} \end{array}$$

where *G*(f^(*M*)^) is some scalar measure of the body load, *n*^(*M*)^ is the number of muscles in the mechanism, and the set of side constraints *f_i_*^(*M*)^ is the force produced by muscle. *C* is a matrix of coefficients depending on current position of body segments, *f* is a vector of unknown forces and *r* consists of external forces and inertia.

#### 2.2.3. Finite Element Analysis Operation

The knee joint cross-sectional images of 2 healthy adult male volunteers participating in this experiment were collected by CT computer scanning. The original three-dimensional image of knee joint in DICOM format was imported into Mimics 10.01 (Materialise, Leuven, Belgium), the tibia was extracted, and the finite element was divided into tetrahedral elements [11], and the tibial model was derived in stl format [12]. After importing stl file into Geomagic studio 12.0 (Geomagic company, Research Triangle Park, NC, USA), the mesh doctor can repair the mesh, fill the holes and repair the sharp corners, and then transfer to ANSYS 19.2 (SwansonAnalysis Inc., Houston, PA, USA) software, simulate the biomechanical experimental model [13], add the maximum stress of knee joint in frontal axis, sagittal axis and vertical axis direction derived from AnyBody 5.2, the Finite Element Analysis was carried out and the data were verified effectively.

### 2.3. Statistical Treatment

SPSS 26.0 was used to process and analyze the data. A single factor analysis of variance and an independent sample *t* test were used to compare the differences of the different indicators. *p* < 0.05 indicated significant differences.

## 3. Results

### 3.1. Kinematic Characteristics of the Different Load Strength Tests

#### 3.1.1. Peak Results of Different Load Intensity Test Angles

**Figure 4:**
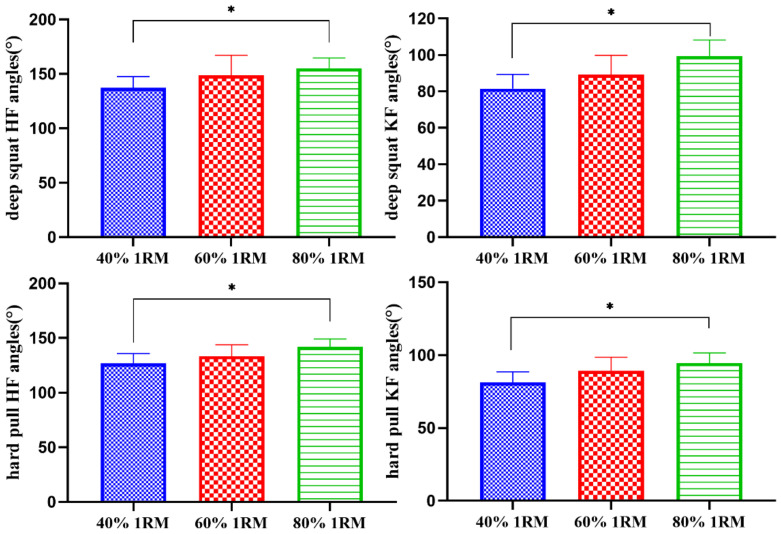
Comparisons of the different load intensity test angles (°). * There was a significant difference between 40% 1RM (One repetition maximum testing) and 80% 1RM (*p* < 0.05).

As shown in Table 1 and Figure 4, under the loads of 40% 1RM and 80% 1RM, there were significant differences in the angles of the deep squat hip flexion, the deep squat knee flexion, the hard pull hip flexion and the knee flexion (*p* < 0.05).

#### 3.1.2. Peak Characteristics of the Angular Velocity as Measured by Different Load Intensities

**Figure 5:**
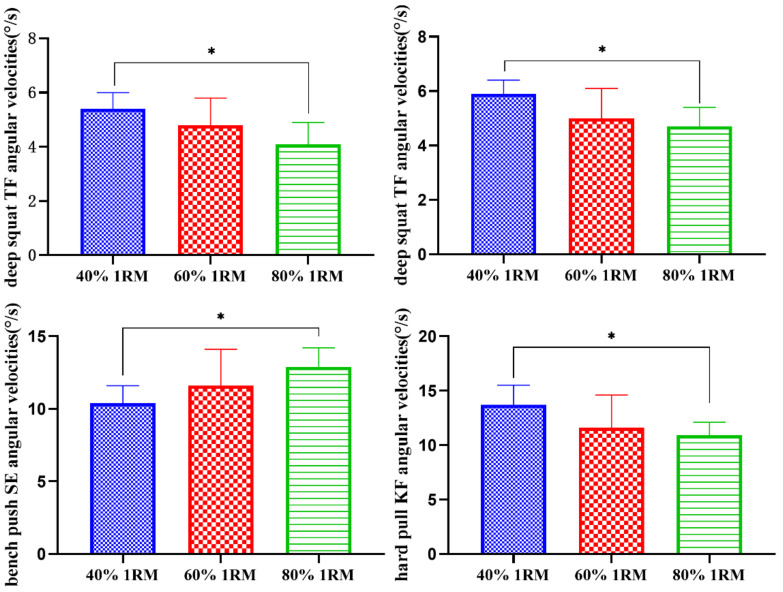
Comparisons of the different load intensity test angular velocities (°/s). * There was a significant difference between 40% 1RM and 80% 1RM (*p* < 0.05).

As shown in Table 2 and Figure 5, under the loads of 40% 1RM and 80% 1RM, there were significant differences in the angular velocities of the deep squat trunk flexion and trunk extension, the bench press shoulder extension and the hard pull knee flexion (*p* < 0.05).

#### 3.1.3. Peak Angular Acceleration Results of the Different Load Intensities

**Figure 6:**
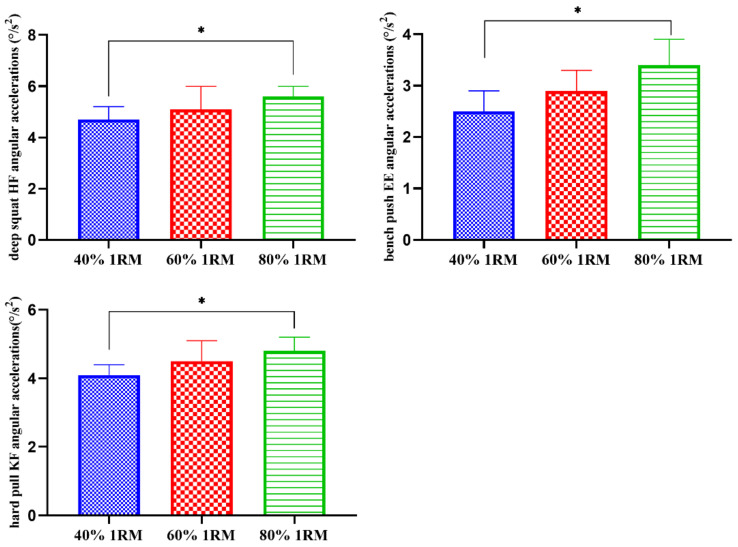
Comparisons of the different load intensity test angular accelerations (°/s^2^). * There was a significant difference between 40% 1RM and 80% 1RM (*p* < 0.05).

As shown in Table 3 and Figure 6, under the loads of 40% 1RM and 80% 1RM, there were significant differences in the angular accelerations of the deep squat hip flexion, the bench press elbow extension, and the hard pull knee flexion (*p* < 0.05).

### 3.2. Dynamic Characteristics of Different Load Strength Tests

#### 3.2.1. Stress of Different Load Strength Tests

**Figure 7:**
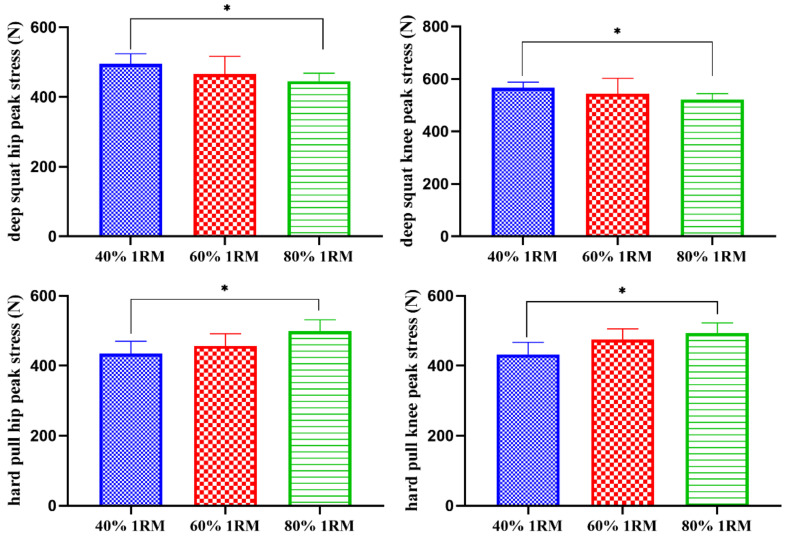
Comparisons of the different load intensity test peak stress (N). * There was a significant difference between 40% 1RM and 80% 1RM (*p* < 0.05).

As shown in Table 4 and Figure 7, under the loads of 40% 1RM and 80% 1RM, there were significant differences in the peak stress of the deep squat hip joint, the deep squat knee joint, the hard pull hip joint and the hard pull knee joint (*p* < 0.05).

#### 3.2.2. Torque of Different Load Strength Tests

**Figure 8:**
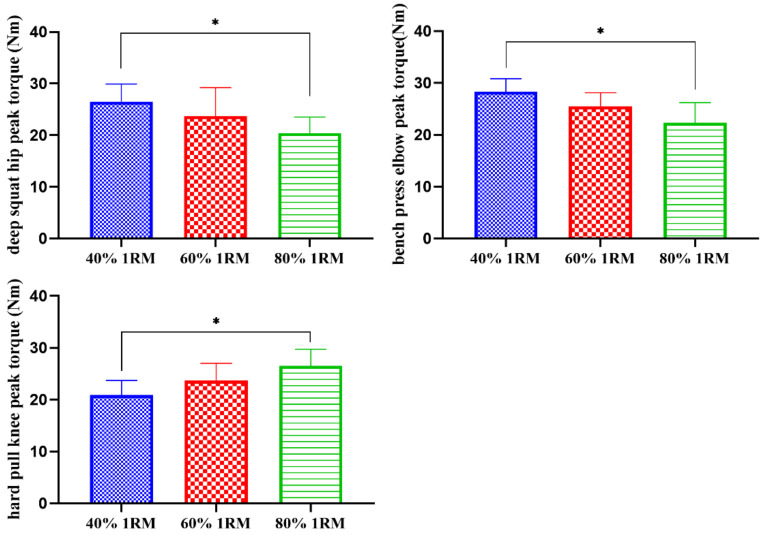
Comparisons of the different load intensity test peak muscle strength (N). * There was a significant difference between 40% 1RM and 80% 1RM (*p* < 0.05).

As shown in Table 5 and Figure 8, under the loads of 40% 1RM and 80% 1RM, there were significant differences in the peak torques of the deep squat hip joint, the bench press elbow joint and the hard pull knee joint (*p* < 0.05).

#### 3.2.3. AnyBody Simulation Results

**Figure 9:**
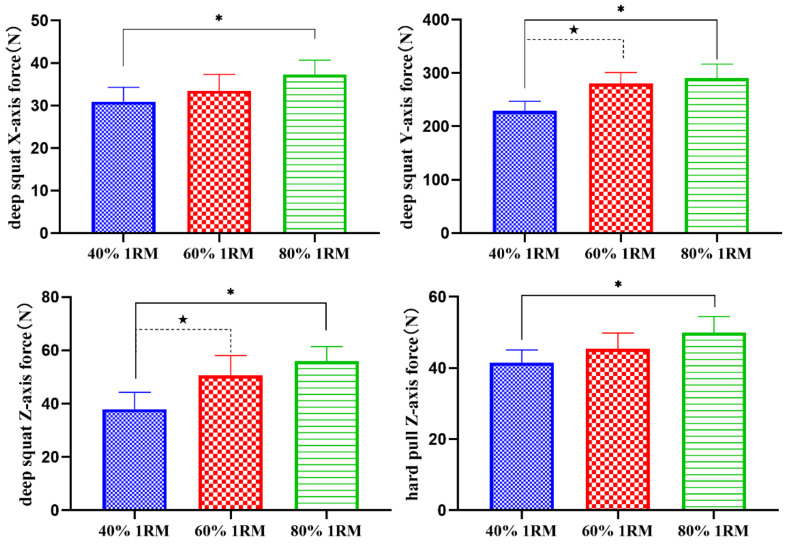
Comparisons of the different force on knee joint (N). * There was a significant difference between 40% 1RM and 80% 1RM (*p* < 0.05). ^★^ There was a significant difference between 40% 1RM and 60% 1RM (*p* < 0.05).

As shown in Table 6 and Figure 9, under the loads of 40% 1RM and 80% 1RM, there were significant differences in the *x*-axis force of the deep squat knee joint, the *y*-axis force of the deep squat knee joint, the *z*-axis force of the deep squat knee joint and the hard pull knee joint (*p* < 0.05), under the loads of 40% 1RM and 60% 1RM, there were significant differences in the *y*-axis force of the deep squat knee joint, the *z*-axis force of the deep squat knee joint (*p* < 0.05).

#### 3.2.4. Finite Element Analysis Results

**Figure 10:**
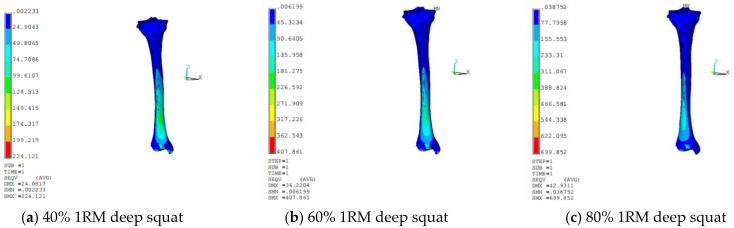
Cloud chart of tibia stress distribution of deep squat.

**Figure 11:**
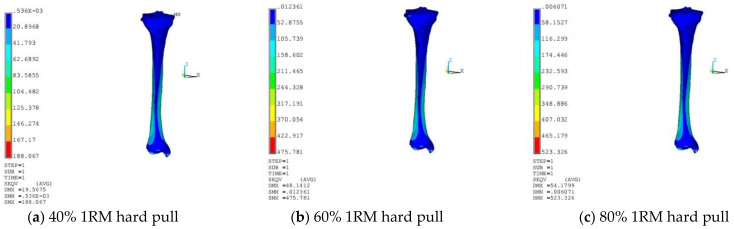
Cloud chart of tibia stress distribution of hard pull.

Figure 10 and Figure 11 are the distribution of Solution Max (SMX) and Solution Min (SMN) in three directions of deep squatting and hard pull, the change of deep squat stress is greater than hard pull, with the increase of load, the stress area of cortical bone increased.

## 4. Discussion

The wearable device can record the data accuracy of the motion test information, can monitor the path target parameters, can adjust for the angles and calculation accuracy. Research involving resistance training is of great significance, which can stimulate muscle growth. Table 1, Table 2 and Table 3 calculates the characteristics of the kinematic parameters, and Table 4, Table 5 and Table 6 calculates the characteristics of the dynamic parameters.

Squatting is a movement in which the body speed is added to the vertical track. To ensure that the core area is tight, the human body relies on the ground reaction force to complete the knee and hip extension actions, in order to accelerate muscle growth and to improve the metabolic rate [14]. Walilko et al. [15] found that the deep squat can improve hamstring muscle flexibility, bilateral symmetry and balance ability, which is an important method for developing the maximum strength of the lower limbs. This study is consistent with these results. When squatting, the hip joint is flexed and extended in the sagittal plane. The bending of the hip joint and the knee joint changes the center of gravity of the trunk’s forward tilt. When lifting the bar, the tendon senses the stimulus feedback and receives feedback from the nervous system, thus allowing for the simultaneous control of the muscles. The muscle is then stimulated and quickly reacts to complete deep squat.

The bench press is an important method of upper limb strength training [16]. Sakamoto et al. [17] found that the muscle activation and fatigue degree are different when different speeds of the bench press. Yamanaka et al. [18] found that, if the wrist posture is wrong, then small muscle will weaken the weight of the bench press. Therefore, the feet cannot be lifted of the ground to prevent the human body from losing the power of the feet. Ramos et al. [19] studied the bench press tests of 20–70% 1RM of the subjects, and they found that there was no significant difference between adjacent loads and that the optimal output power was 1RM of 20–40%. Similar to the results of Thiesfield and Ramos, we found that the optimal power of the bench press was 40% 1RM, which indicates that resistance exercise has a consistent influence on human motion performance and functional mode.

The hard pull mainly focuses on back and hip leg training. Suchomel et al. [20] studied the hard pullmaneuvers of college students to make their back arches straight. With the help of the flexibility and force detection of the thigh, the back muscle group maintained static contraction. It was found that the main muscle groups involved in the hard pull were the quadriceps femoris, the hamstring muscle, the erector spinalis and the trapezius muscle. It was also found that 70–80% of the training limit weight can produce the best training effect. The results showed that the maximum peak force appeared at the 70–80% 1RM load intensity. The results of this study showed that the hard pull force of 80% 1RM are the highest, which indicates that the best explosive force training with this weight can produce significant effects. Therefore, it is suggested that people with certain strength training bases should perform explosive force training with an 80% 1RM load in the future.

The knee joint provides support when people walk, sit, squat. In the process of knee joint modeling, the distribution of trabeculae and bone cortex is positively correlated with stress. The denser the trabeculae is, the more active osteoblasts are and the greater the stress is. The conventional material properties of knee joint are cancellous bone and dense bone, and Poisson ratio and elastic modulus are given in the same direction. Based on the different mechanical properties of bone region during deep squat and hard pull, the results are different. The stress distribution of the above model shows the relationship between the force and the load weight, which indicates that the external force is the main factor of bone change.

In addition, previous studies have focused on neuromuscular characteristics, which can be combined with kinematics, dynamics, and neuroelectromyography parameters to verify data consistency, It has been observed that different levels of research objects muscle strength levels were different, which may cause errors. Additionally, the lack of comparisons of different load cases at different speeds will have a certain impact on individual differences. Based on the improvement of anti-interference, it is necessary to perform joint research on neuromuscular characteristics.

## 5. Conclusions

The hip joint and knee joint exhibit increased angles, angular velocities and angular accelerations of the deep squat and the hard pull, whereas the elbow joint exhibited increased angles, angular velocities and angular accelerations of the bench press. The best force and power of the deep squat elbow joint, the hip joint and the knee joint is 40% 1RM, that of the bench press is 40% 1RM and that of the hard pull is 80% 1RM. The main force producing portions of the deep squat are the hip joint and the knee joint, the dominant force producing portions of the bench press are the shoulder joint and the elbow joint, and the main force generating portions of the hard pull are the hip joint, the knee joint and the trunk. The external force is the main factor of bone change. The mechanical characteristics of knee joint can be simulated after the Finite Element Analysis and the simulation of AnyBody are verified.

## Figures and Tables

**Table 1:** Comparisons of the peak values of the different load intensity test angles (°).

	Deep Squat			Bench Press			Hard Pull		
	40% 1RM	60% 1RM	80% 1RM	40% 1RM	60% 1RM	80% 1RM	40% 1RM	60% 1RM	80% 1RM
CF	13.5 ± 1.0	13.5 ± 1.1	13.6 ± 1.6	1.4 ± 0.1	1.4 ± 0.3	1.49 ± 0.09	13.1 ± 4.3	13.3 ± 5.6	13.3 ± 6.5
CE	28.5 ± 5.6	28.7 ± 5.4	28.9 ± 6.3	1.3 ± 0.2	1.3 ± 0.2	1.32 ± 0.29	26.0 ± 8.2	26.1 ± 8.0	26.1 ± 7.3
TF	24.7 ± 3.7	24.8 ± 3.2	24.8 ± 3.0	1.8 ± 0.2	1.8 ± 0.2	1.92 ± 0.2	20.8 ± 2.4	21.3 ± 3.1	21.6 ± 4.2
TE	35.6 ± 4.8	36.7 ± 5.6	36.9 ± 5.2	1.5 ± 0.4	1.5 ± 0.3	1.64 ± 0.6	33.5 ± 3.9	33.6 ± 4.5	33.7 ± 3.6
SF	55.6 ± 10.2	55.6 ± 6.3	55.7 ± 7.8	16.2 ± 9.0	17.8 ± 9.9	17.9 ± 8.9	50.2 ± 5.6	52.6 ± 14.3	53.2 ± 8.4
SE	53.0 ± 5.9	55.4 ± 4.8	55.6 ± 4.3	16.2 ± 10.6	16.3 ± 8.0	17.3 ± 2.1	53.3 ± 1.5	53.4 ± 1.0	53.4 ± 1.2
EF	45.5 ± 8.3	45.6 ± 7.5	45.7 ± 7.9	168.0 ± 18.5	168.2 ± 6.6	168.2 ± 9.3	40.3 ± 5.9	40.3 ± 6.6	40.3 ± 5.0
EE	43.0 ± 10.2	43.1 ± 6.7	43.2 ± 8.0	160.2 ± 17.2	163.5 ± 7.4	175.7 ± 8.9	49.7 ± 8.5	49.2 ± 8.1	49.5 ± 8.6
HF	137.2 ± 10.3	148.7 ± 18.6	155.3 ± 9.4 *	35.8 ± 6.9	39.5 ± 8.6	40.8 ± 8.6	126.8 ± 8.9	133.4 ± 10.3	141.9 ± 7.2 *
HE	136.7 ± 15.6	137.8 ± 12.1	138.9 ± 8.3	30.5 ± 6.3	32.8 ± 5.0	33.7 ± 6.9	130.4 ± 9.8	133.6 ± 10.2	142.8 ± 9.3
KF	81.6 ± 7.8	89.3 ± 10.6	99.4 ± 8.8 *	34.9 ± 9.5	36.3 ± 10.3	37.8 ± 8.6	81.3 ± 7.3	89.2 ± 9.4	94.5 ± 6.9 *
KE	82.3 ± 10.2	85.6 ± 15.5	86.3 ± 9.7	35.2 ± 8.5	36.4 ± 7.2	35.9 ± 8.1	82.1 ± 10.9	84.7 ± 8.3	85.4 ± 10.2

Notes: CF: Cervical Flexion; CE: Cervical Extension; TF: Trunk Flexion; TE: Trunk Extension; SF: Shoulder Flexion; SE: Shoulder Extension; EF: Elbow Flexion; EE: Elbow Extension; HF: Hip Flexion; HE: Hip Extension; KF: Knee Flexion; KE: Knee Extension. * There was a significant difference between 40% 1RM and 80% 1RM (*p* < 0.05).

**Table 2:** Comparisons of the peak values of different load intensity test angular velocities (°/s).

	Deep Squat			Bench Press			Hard Pull		
	40% 1RM	60% 1RM	80% 1RM	40% 1RM	60% 1RM	80% 1RM	40% 1RM	60% 1RM	80% 1RM
CF	3.0 ± 0.5	2.6 ± 0.6	2.8 ± 0.4	3.3 ± 0.9	3.5 ± 0.9	3.8 ± 0.9	2.4 ± 0.6	2.3 ± 0.6	2.8 ± 0.6
CE	3.5 ± 0.8	2.7 ± 0.8	3.0 ± 0.5	3.8 ± 0.8	3.6 ± 0.8	3.5 ± 0.9	2.3 ± 0.7	2.6 ± 0.8	2.6 ± 0.9
TF	5.4 ± 0.6	4.8 ± 1.0	4.1 ± 0.8 *	1.8 ± 0.9	1.9 ± 0.8	2.0 ± 1.0	10.4 ± 2.0	11.5 ± 2.1	11.1 ± 2.0
TE	5.9 ± 0.5	5.0 ± 1.1	4.7 ± 0.7 *	1.9 ± 0.8	1.9 ± 0.9	2.1 ± 0.9	10.1 ± 2.7	11.9 ± 2.0	11.6 ± 2.8
SF	10.1 ± 2.0	10.2 ± 2.0	10.1 ± 2.0	10.0 ± 2.3	11.6 ± 2.6	12.8 ± 3.5	13.4 ± 3.0	13.2 ± 2.1	13.2 ± 3.3
SE	10.2 ± 2.4	10.1 ± 2.4	9.7 ± 2.3	10.4 ± 1.2	11.6 ± 2.5	12.9 ± 1.3 *	13.3 ± 3.3	13.3 ± 2.0	13.5 ± 3.1
EF	5.9 ± 1.2	5.7 ± 1.1	5.6 ± 1.2	9.6 ± 2.7	9.6 ± 1.5	10.7 ± 2.6	12.2 ± 3.4	11.0 ± 2.1	12.2 ± 1.5
EE	5.9 ± 1.0	5.7 ± 1.0	5.7 ± 1.0	10.4 ± 2.3	10.5 ± 2.4	10.2 ± 2.4	12.9 ± 3.2	11.1 ± 2.0	12.1 ± 1.0
HF	10.5 ± 2.5	11.5 ± 3.6	12.2 ± 3.6	7.3 ± 2.9	8.3 ± 2.0	8.4 ± 3.1	10.2 ± 3.9	10.2 ± 3.5	10.3 ± 3.5
HE	11.0 ± 2.6	11.42 ± 3.4	12.3 ± 3.3	6.4 ± 2.3	7.3 ± 2.3	7.6 ± 2.4	9.4 ± 3.2	10.0 ± 3.8	10.6 ± 3.2
KF	12.4 ± 3.7	12.9 ± 3.7	13.6 ± 3.8	7.43 ± 2.4	7.4 ± 2.5	7.3 ± 2.4	13.7 ± 1.8	11.6 ± 3.0	10.9 ± 1.2 *
KE	12.5 ± 3.3	12.8 ± 3.5	12.9 ± 3.5	7.4 ± 2.0	7.5 ± 2.0	7.5 ± 2.0	18.1 ± 3.5	17.2 ± 3.3	16.2 ± 2.1

Notes: CF: Cervical Flexion; CE: Cervical Extension; TF: Trunk Flexion; TE: Trunk Extension; SF: Shoulder Flexion; SE: Shoulder Extension; EF: Elbow Flexion; EE: Elbow Extension; HF: Hip Flexion; HE: Hip Extension; KF: Knee Flexion; KE: Knee Extension. * There was a significant difference between 40% 1RM and 80% 1RM (*p* < 0.05).

**Table 3:** Comparisons of the peak values of different load intensity test angular accelerations (°/s^2^).

	Deep Squat			Bench Press			Hard Pull		
	40% 1RM	60% 1RM	80% 1RM	40% 1RM	60% 1RM	80% 1RM	40% 1RM	60% 1RM	80% 1RM
CF	0.2 ± 0.0	0.2 ± 0.0	0.2 ± 0.0	0.1 ± 0.0	0.1 ± 0.0	0.1 ± 0.0	0.1 ± 0.0	0.1 ± 0.0	0.1 ± 0.0
CE	1.2 ± 0.2	1.2 ± 0.2	1.3 ± 0.2	0.1 ± 0.0	0.1 ± 0.0	0.1 ± 0.0	0.3 ± 0.0	0.3 ± 0.1	0.3 ± 0.1
TF	0.2 ± 0.0	0.2 ± 0.0	0.2 ± 0.1	0.1 ± 0.0	0.1 ± 0.0	0.1 ± 0.0	0.2 ± 0.1	0.2 ± 0.1	0.2 ± 0.1
TE	0.1 ± 0.0	0.1 ± 0.0	0.1 ± 0.0	0.4 ± 0.1	0.4 ± 0.1	0.4 ± 0.1	1.6 ± 0.3	1.6 ± 0.3	1.6 ± 0.2
SF	0.3 ± 0.1	0.3 ± 0.0	0.3 ± 0.1	0.9 ± 0.5	0.9 ± 0.4	0.9 ± 0.4	0.2 ± 0.1	0.2 ± 0.1	0.2 ± 0.1
SE	2.7 ± 0.7	2.8 ± 0.8	2.8 ± 0.8	0.9 ± 0.2	0.9 ± 0.2	1.0 ± 0.3	0.2 ± 0.0	0.2 ± 0.0	0.2 ± 0.0
EF	1.8 ± 0.4	1.9 ± 0.6	1.9 ± 0.5	2.3 ± 1.5	2.5 ± 1.3	2.6 ± 0.4	0.1 ± 0.0	0.1 ± 0.0	0.1 ± 0.0
EE	0.2 ± 0.1	0.2 ± 0.1	0.2 ± 0.1	2.5 ± 0.4	2.9 ± 0.4	3.4 ± 0.5 *	0.2 ± 0.0	0.2 ± 0.0	0.2 ± 0.0
HF	4.7 ± 0.5	5.1 ± 0.9	5.6 ± 0.4 *	0.4 ± 0.1	0.4 ± 0.1	0.4 ± 0.2	2.2 ± 0.3	2.2 ± 0.3	2.3 ± 0.5
HE	4.6 ± 0.9	4.6 ± 0.8	4.6 ± 0.9	0.1 ± 0.0	0.1 ± 0.0	0.1 ± 0.0	3.9 ± 1.0	3.9 ± 1.2	4.0 ± 1.3
KF	4.2 ± 1.5	4.3 ± 1.0	4.3 ± 1.2	0.1 ± 0.0	0.1 ± 0.0	0.1 ± 0.0	4.1 ± 0.3	4.5 ± 0.6	4.8 ± 0.4 *
KE	4.4 ± 1.1	4.4 ± 0.7	4.6 ± 0.9	0.2 ± 0.1	0.2 ± 0.1	0.2 ± 0.1	3.9 ± 0.9	4.0 ± 0.8	4.1 ± 1.2

Notes: CF: Cervical Flexion; CE: Cervical Extension; TF: Trunk Flexion; TE: Trunk Extension; SF: Shoulder Flexion; SE: Shoulder Extension; EF: Elbow Flexion; EE: Elbow Extension; HF: Hip Flexion; HE: Hip Extension; KF: Knee Flexion; KE: Knee Extension. * There was a significant difference between 40% 1RM and 80% 1RM (*p* < 0.05).

**Table 4:** Comparisons of peak stress in different load strength tests (N).

	Deep Squat			Bench Press			Hard Pull		
	40% 1RM	60% 1RM	80% 1RM	40% 1RM	60% 1RM	80% 1RM	40% 1RM	60% 1RM	80% 1RM
Cervical	32.6 ± 5.8	35.7 ± 6.4	35.5 ± 7.6	13.6 ± 4.2	12.1 ± 3.4	11.5 ± 4.6	17.6 ± 4.3	18.4 ± 3.6	18.9 ± 4.7
Trunk	165.3 ± 10.2	167.4 ± 13.6	167.7 ± 9.3	94.4 ± 16.5	93.5 ± 19.1	91.3 ± 20.4	371.3 ± 56.3	378.3 ± 67.9	382.1 ± 71.4
Shoulder	297.5 ± 52.3	295.8 ± 54.1	298.7 ± 55.6	575.7 ± 50.9	573.6 ± 48.5	561.9 ± 52.6	162.9 ± 74.3	163.5 ± 86.4	169.5 ± 10.9
Elbow	228.9 ± 75.3	223.6 ± 87.4	221.4 ± 72.3	439.6 ± 42.7	436.7 ± 53.4	430.2 ± 46.2	56.3 ± 10.9	58.6 ± 14.8	59.3 ± 9.6
Hip	495.3 ± 28.9	465.9 ± 50.6	445.6 ± 22.5 *	151.5 ± 23.2	147.3 ± 38.2	145.6 ± 25.5	434.6 ± 35.2	456.7 ± 34.3	498.5 ± 32.6 *
Knee	566.5 ± 21.3	543.6 ± 58.7	520.8 ± 23.4 *	120.6 ± 25.6	113.7 ± 32.5	110.8 ± 35.7	432.3 ± 34.6	475.4 ± 29.7	493.2 ± 29.1 *

Notes: * There was a significant difference between 40% 1RM and 80% 1RM (*p* < 0.05).

**Table 5:** Comparisons of peak torques in different load strength tests (Nm).

	Deep Squat			Bench Press			Hard Pull		
	40% 1RM	60% 1RM	80% 1RM	40% 1RM	60% 1RM	80% 1RM	40% 1RM	60% 1RM	80% 1RM
Cervical	5.6 ± 1.5	5.7 ± 1.6	5.8 ± 1.0	4.8 ± 1.2	4.7 ± 0.8	4.3 ± 1.6	5.3 ± 1.8	5.9 ± 1.6	5.9 ± 1.5
Trunk	8.3 ± 2.8	9.4 ± 3.5	9.6 ± 5.9	13.1 ± 3.8	12.8 ± 4.2	11.6 ± 3.4	19.7 ± 4.3	17.4 ± 5.8	18.5 ± 6.7
Shoulder	13.5 ± 4.8	13.8 ± 4.5	11.6 ± 5.9	27.6 ± 1.3	23.7 ± 1.2	20.9 ± 1.1	13.2 ± 2.7	13.8 ± 2.3	13.9 ± 2.1
Elbow	14.2 ± 5.6	14.3 ± 4.2	14.3 ± 6.3	28.3 ± 2.5	25.5 ± 2.6	22.3 ± 3.9 *	10.7 ± 4.6	11.7 ± 4.2	11.8 ± 4.2
Hip	26.5 ± 3.4	23.7 ± 5.5	20.4 ± 3.1 *	12.3 ± 2.6	10.5 ± 3.4	10.2 ± 4.0	23.4 ± 7.9	23.6 ± 6.8	23.1 ± 7.7
Knee	27.1 ± 8.3	25.9 ± 7.6	23.9 ± 8.3	13.8 ± 5.9	12.9 ± 3.5	12.6 ± 4.3	20.9 ± 2.8	23.7 ± 3.3	26.5 ± 3.2 *

Notes: * There was a significant difference between 40% 1RM and 80% 1RM (*p* < 0.05).

**Table 6:** Force on knee joint in different directions (N).

	Deep Squat			Hard Pull		
	40% 1RM	60% 1RM	80% 1RM	40% 1RM	60% 1RM	80% 1RM
*x*-axis force	30.93 ± 3.40	33.46 ± 3.91	37.32 ± 3.36 *	9.25 ± 3.39	10.65 ± 1.42	12.84 ± 3.20
*y*-axis force	229.25 ± 17.62	280.37 ± 20.38 ^★^	290.36 ± 26.53 *	453.13 ± 30.46	489.17 ± 40.31	517.21 ± 40.05
*z*-axis force	37.91 ± 6.34	50.58 ± 7.44 ^★^	55.91 ± 5.46 *	41.54 ± 3.52	45.42 ± 4.38	49.93 ± 4.56 *

Notes: * There was a significant difference between 40% 1RM and 80% 1RM (*P* < 0.05); ^★^ There was a significant difference between 40% 1RM and 60% 1RM (*p* < 0.05).

## Data Availability

The data presented in this study are available on request from the corresponding author.

## References

[1] Rosenberger M.E., Buman M.P., Haskell W.L., McConnell M.V., Carstensen L.L. (2016). Twenty-four hours of sleep, sedentary behavior, and physical activity with nine wearable devices. Med. Sci. Sports Exerc..

[2] Jung J., Leung W.C., Case L.K., Yun J. (2018). Not all are created equal: A meta-analysis of wearable devices for tracking physical activity. Med. Sci. Sports Exerc..

[3] Plasqui G., Bonomi A.G., Westerterp K.R. (2013). Daily physical activity assessment with accelerometers: New insights and validation studies. Obes. Rev..

[4] Samsonova A.G., Ponomarev G.N., Tsipin L.L. (2018). Strength training biomechanics concept for athletic training systems. Teor. Prak. Fiz. Kult..

[5] Mcbride J.M., Skinner J.W., Schafer P.C., Haines T.L., Kirby T.J. (2010). Comparison of kinetic variables and muscle activity during a squat vs. a box squat. J. Strength Cond. Res..

[6] Kraemer W.J., Ratamess N.A. (2004). Fundamentals of resistance training: Progression and exercise prescription. Med. Sci. Sports Exerc..

[7] Collins J., Longhurst G., Roschel H., Gualano B. (2016). Resistance training and co-supplementation with creatine and protein in older subjects with frailty. J. Frailty Aging.

[8] Hughes L.J., Jeremiah J.P., Scott B.R. (2020). Load-velocity relationship 1RM predictions: A comparison of Smith machine and free-weight exercise. J. Sports Sci..

[9] Rasmussen P., Damsgaard M., Voigt M. (2001). Muscle recruitment by the min/max criterion—A comparative numerical study. J. Biomech..

[10] Saraswat P., Andersen M.S., MacWilliams B.A. (2010). A musculoskeletal foot model for clinical gait analysis. J. Biomech..

[11] Small S.R., Berend M.E., Rogge R.D., Archer D.B., Kingman A.L., Ritter M.A. (2013). Tibial loading after UKA: Evaluation of tibial slope, resection depth, medial shift and component rotation. J. Arthroplast..

[12] Hopkins A.R., Mew A.M., Rodriguez-y-Baena F., Taylor M. (2010). Finite element analysis of unicompartmental knee arthroplasty. Med. Eng. Phys..

[13] Burkhart T.A., Andrews D.M., Dunning C.E. (2013). Finite element modeling mesh quality, energy balance and validation methods: A review with recommendations associated with the modeling of bone tissue. J. Biomech..

[14] Faigenbaum A.D., Kraemer J.W., Blimkie J.R., Jeffreys I., Micheli L.J., Nitka M., Rowland T.W. (2009). Youth resistance training: Updated position statement paper from the national strength and conditioning association. J. Strength Cond. Res..

[15] Walilko T.J., Viano D.C., Bir C.A. (2005). Biomechanics of the head for Olympic boxer punches to the face. Br. J. Sports Med..

[16] Calatayud J., Vinstrup J., Jakobsen M.D., Sundstrup E., Colado J.C., Andersen L.L. (2017). Influence of different attentional focus on EMG amplitude and contraction duration during the bench press at different speeds. J. Sports Sci..

[17] Sakamoto A., Sinclair P.J. (2012). Muscle activations under varying lifting speeds and intensities during bench press. Eur. J. Appl. Physiol..

[18] Yamanaka T., Farley R.S., Caputo J.L. (2012). Occlusion training increases muscular strength in division IA football players. J. Strength Cond. Res..

[19] García-Ramos A., Haff G.G., Padial P., Feriche B. (2016). Optimal load for maximizing upper-body power: Test-retest reproducibility. Isokinet. Exerc. Sci..

[20] Suchomel T.J., Beckham G.K., Wright G.A. (2015). Effect of various loads on the force-time characteristics of the hang high pull. J. Strength Cond. Res..

